# Oxidative stress mediates the apoptosis and epigenetic modification of the Bcl-2 promoter via DNMT1 in a cigarette smoke-induced emphysema model

**DOI:** 10.1186/s12931-020-01495-w

**Published:** 2020-09-03

**Authors:** Huihui Zeng, Tiao Li, Xue He, Shan Cai, Hong Luo, Ping Chen, Yan Chen

**Affiliations:** 1 Department of Pulmonary and Critical Care Medicine, the Second Xiangya Hospital, Central South University, No. 139 Renmin Road, Changsha, 410011 Hunan China; 2 Research Unit of Respiratory Diseases, Central South University, No. 139 Renmin Road, Changsha, 410011 Hunan China; 3 Hunan Centre for Evidence-based Medicine, No. 139 Renmin Road, Changsha, 410011 Hunan China

**Keywords:** Oxidative stress, Apoptosis, Hypermethylation, Bcl-2, DNMT1

## Abstract

**Background:**

Emphysema is a crucial pathological characteristic of chronic obstructive pulmonary disease (COPD). Oxidative stress, apoptosis and epigenetic mechanisms contribute to the pathogenesis of emphysema. However, an attempt to accurately identify whether these mechanisms interact with each other and how they are triggered has never been conducted.

**Method:**

The total reactive oxygen species (ROS) level, pulmonary apoptosis and B-cell lymphoma/leukemia-2 (Bcl-2) expression, an apoptosis regulator, were detected in samples from COPD patients. Bisulfite sequencing PCR (BSP) was conducted to observe the alterations in the methylation of the Bcl-2 promoter in specimens. The dysregulation of DNA methyltransferase enzyme 1 (DNMT1), a vital DNA methyltransferase enzyme, in the lungs of patients was confirmed through western blotting. To find out interactions between oxidative stress and DNA methylation in emphysema, mouse models were built with antioxidant treatment and DNMT1 silencing, and were examined with the pulmonary apoptosis, Bcl-2 and DNMT1 levels, and epigenetic alterations of Bcl-2.

**Results:**

Higher ROS levels and pulmonary apoptosis were observed in COPD patients than in healthy controls. Downregulated Bcl-2 expression with increased promoter methylation and DNMT1 protein expression was found in COPD patients. Antioxidant treatment reduced the level of ROS, DNMT1 protein and emphysematous progression in the smoking models. Following DNMT1 blockade, smoking models showed improved lung function, pulmonary apoptosis, emphysematous progression, and increased Bcl-2 protein level with less promoter methylation than emphysema mice.

**Conclusion:**

Cigarette-induced oxidative stress mediates pulmonary apoptosis and hypermethylation of the Bcl-2 promoter in emphysema models through DNMT1.

## Introduction

Chronic obstructive pulmonary disease (COPD) is a worldwide public health burden because of its high prevalence [1]. A recent study [2] showed that the estimated total number of individuals aged 20 years or older with spirometry-defined COPD in China was 99.9 million in 2015. Cigarette smoking is an established risk factor for COPD, and smoking cessation seems to be the most effective intervention for COPD [1–3]. However, an abundance of studies have demonstrated that pulmonary pathological progress was ongoing and could not be reversed in COPD patients after smoking cessation [4–6]. There are several mechanisms that contribute to the pathogenesis of emphysema, a prominent pathological hallmark of COPD, such as persistent inflammation, proteolytic/anti-proteolytic imbalance, oxidative stress, and apoptosis [4, 7, 8].

Several studies [9–12] have demonstrated that oxidative stress was a critical factor, that links inflammation, excessive matrix proteolysis and direct damage to DNA in emphysema. Our previous study and some others [13–16] showed that oxidative stress played an important role in apoptosis in COPD patients and models. Apoptosis is a highly regulated cell suicide program that can be regulated by B-cell lymphoma/leukemia-2 (Bcl-2) family proteins [17, 18]. We also demonstrated that Bcl-2 protein level was decreased in emphysema models and COPD patients, suggesting that Bcl-2 is involved in COPD pathogenesis [19]. The epigenetic regulation of DNA can stably propagate during a lifetime and might account for ongoing disease progression [20, 21]. Promoter methylation is an emerging and important epigenetic regulatory mechanism, that inhibits target gene transcription and blocks gene expression [22, 23]. Our previous studies [19, 24] found a high methylation status of the whole genome and hypermethylation of the Bcl-2 promoter in COPD patients and emphysema models, and demethylation treatment could protect models from emphysema. DNA methyltransferase 1 (DNMT1) is regarded as the primary methyltransferase, contributing to de novo DNA methylation and maintenance [25]. Based on the above studies, we postulated that cigarette smoke (CS)-induced oxidative stress mediates apoptosis and Bcl-2 methylation via DNMT1 in emphysema.

## Results

### Demographic, pulmonary function and morphological findings in COPD patients

We have collected 27patients, who were underwent the excision of peripheral solitary pulmonary nodule or pulmonary lesion. Nine patients are normal nonsmokers, and 9 cases were non-COPD smokers. The last 9 cases were smokers with spirometry-defined COPD. There was no significant difference in the gender ration or age among the three groups (Table 1). The smoking history of non-COPD smokers and COPD smokers was analyzed, and no difference in smoking history was found between the two groups (*P* = 0.23, Table 1).

**Table 1:** Anthropometric characteristics, spirometry values of the subjects

	Nonsmoker *n* = 9	Non-COPD Smoker *n* = 9	COPD smoker *n* = 9	*P* value
Gender (M/n)	66.7% (6/9)	77.8% (7/9)	77.8% (7/9)	0.825 ^a^
Age (years)	56.22 ± 10.99	57.44 ± 5.72	57.67 ± 6.50	0.92^b^
Smoking history (pack-year)	0	40.33 ± 13.61	39.56 ± 14.87	0.23^c^
FEV_1_/Pre (%)	85.11 ± 3.82	85.56 ± 4.22	71.78 ± 3.96	<0.01^b^
FEV_1_/FVC (%)	81.33 ± 5.57	81.11 ± 5.82	63.44 ± 3.50	<0.01^b^

^a^ P by Chi-square test, ^b^P by one-way ANOVA test, and ^c^ P by Student’s t test

**Fig. 1:**
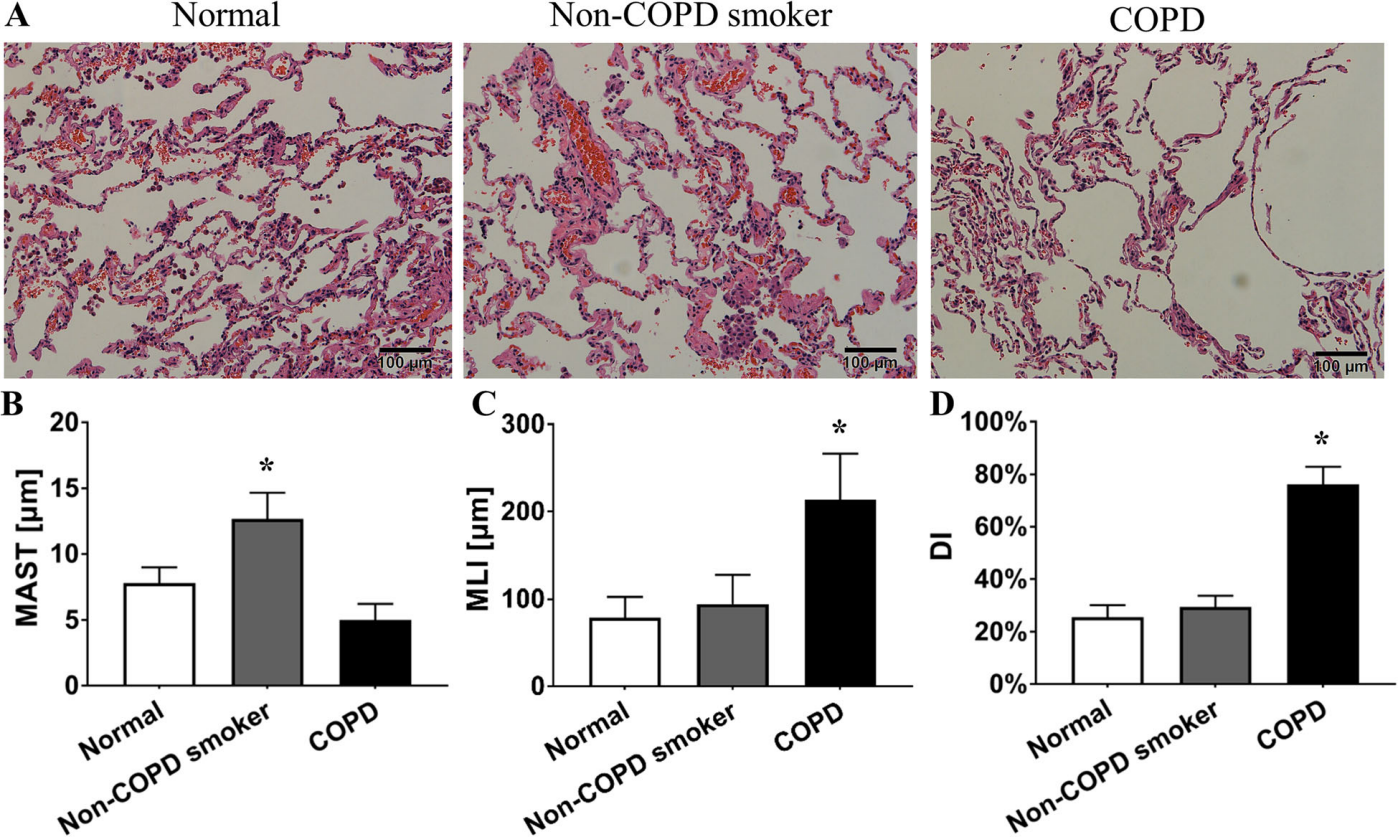
Histological alterations in COPD smoker lungs. **a** Lungs from normal (nonsmokers), non-COPD smokers and COPD smokers stained with HE (100×). **b**, **c**, **d** MAST, MLI and DI in normal (nonsmokers), non-COPD smokers and COPD smokers. Non-COPD smokers did not show an emphysematous change, but had a thicker alveolar septum, MAST (μm), than nonsmokers (12.67 ± 2.00 vs. 7.78 ± 1.20, *P <* 0.01 by one-way ANOVA and LSD test). However, the COPD group had a thinner MAST (μm) than the nonsmoker group (5.00 ± 1.22 vs. 7.78 ± 1.20, *P* < 0.01 by one-way ANOVA and LSD test). COPD patients showed significant emphysematous disorder with a greater MLI (μm) and DI (%) than nonsmokers (213.33 ± 53.15 vs. 78.89 ± 23.69, *P <* 0.01 by Kruskal-Wallis test, and 76.22 ± 6.67 vs. 25.56 ± 4.67, *P <* 0.01 by one-way ANOVA and LSD test, respectively). *P < 0.05 vs. the nonsmoker group. Data in (**b**), (**c**), and (**d**) represent the means ± SD

Compared with the other two groups, the COPD smokers showed destroyed pulmonary architecture and function (*P* < 0.05 by one-way ANOVA and least significant difference (LSD) test, Table 1 and Fig. 1a). Histologically, the nonsmokers presented a well-fixed normal pulmonary parenchyma with normal airways (Fig. 1a). Non-COPD smokers did not exhibit emphysematous changes, but presented a thicker alveolar septum than nonsmokers (mean alveolar septal thickness, MAST; Fig. 1b). COPD patients showed significant emphysematous disorder with an increased mean linear intercept (MLI) and destructive index (DI; Fig. 1c, d).

### Aggravated pulmonary apoptosis and oxidative stress in the lungs of COPD patients

**Fig. 2:**
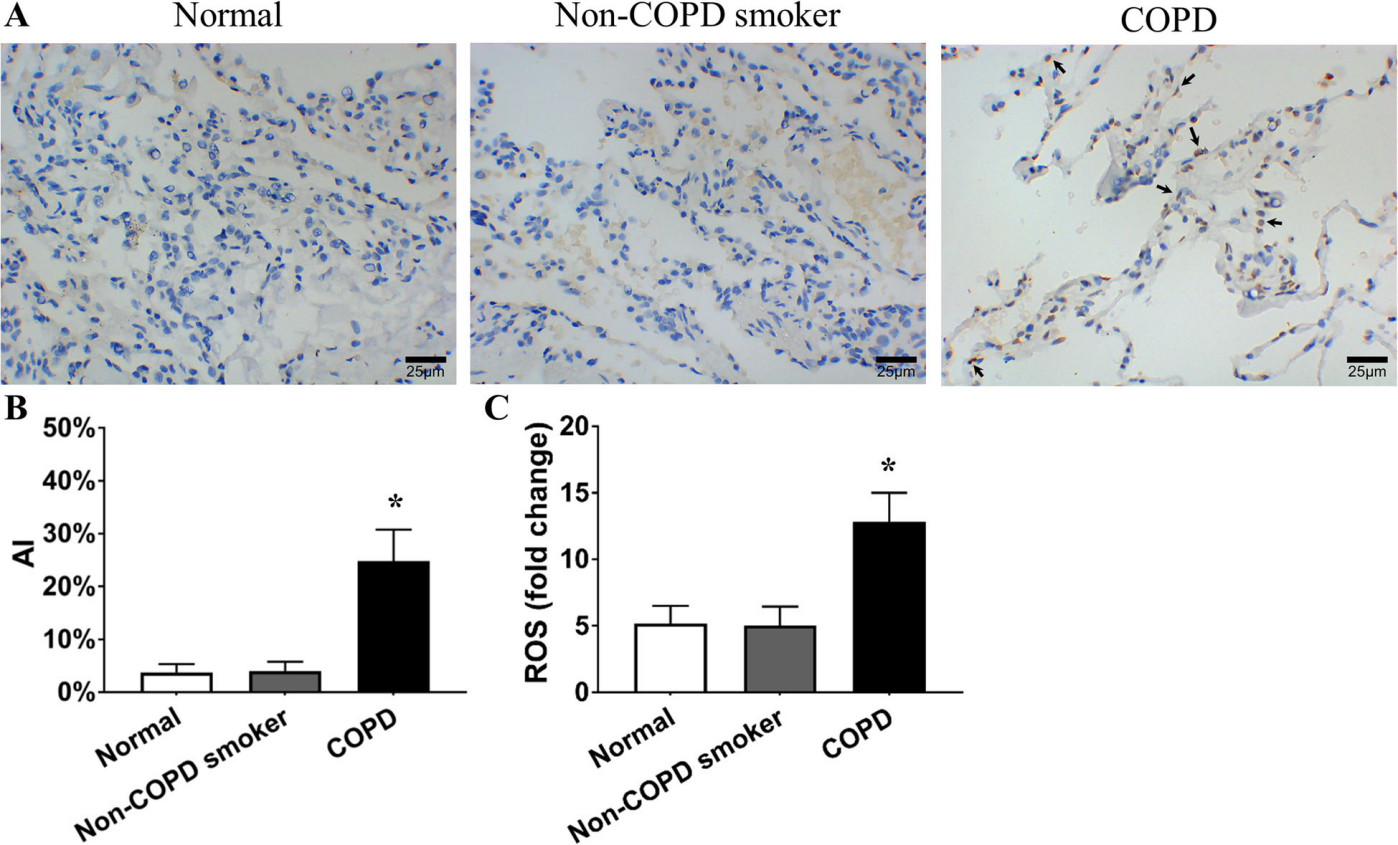
Increased apoptosis and oxidative stress in COPD patient lungs. **a** TUNEL staining was conducted in lung tissue samples from normal (nonsmokers), non-COPD smokers and COPD patients (400×). **b** AI was accounted for in the normal (nonsmoker), non-COPD smoker and COPD groups. → the TUNEL-positive nuclei. Compared to that in the lungs of nonsmokers and smokers without COPD, the AI (%) was increased in the lungs of COPD patients (3.73 ± 1.57 and 3.99 ± 1.75 vs. 24.79 ± 5.95, *P <* 0.01 by Kruskal-Wallis test). Interestingly, there was no difference in pulmonary AI between the nonsmokers and smokers without COPD (*P* = 0.976 by Kruskal-Wallis test). **c** Increased oxidative stress in the lungs of COPD patients. The level of ROS (RFU) in tissues from COPD patients was higher than that in tissues from nonsmokers (12.84 ± 2.16 vs. 5.17 ± 1.33, *P <* 0.01 by one-way ANOVA and LSD test), indicating that there was a higher oxidant burden in COPD patients. In contrast, the smokers without COPD did not show a significant change in ROS levels (5.03 ± 1.44 vs. 5.17 ± 1.33, *P* = 0.861 by one-way ANOVA and LSD test). *P < 0.05 vs. the normal group. Data in (**b**) and (**c**) represent the means ± SD

Compared to that in the lungs of nonsmokers and smokers without COPD, the apoptosis index (AI) was increased in the lungs of COPD patients (Fig. 2a, b). Interestingly, there was no difference in the pulmonary AI (%) between the nonsmokers and smokers without COPD (3.73 ± 1.57 vs. 3.99 ± 1.75, *P* < 0.01 by the Kruskal-Wallis test, Fig. 2a, b). This finding suggests that CS is not the only reason for apoptosis in COPD, and there might be mechanisms beyond direct CS damage. The level of reactive oxygen species (ROS) in tissues from COPD patients was higher than that in tissues from nonsmokers (Fig. 2c), indicating that there was a higher oxidant burden in COPD. In contrast, the smokers without COPD did not show a significant change in ROS levels (Fig. 2c).

### Dysregulation of Bcl-2 and DNMT1 protein levels in lungs from COPD patients

**Fig. 3:**
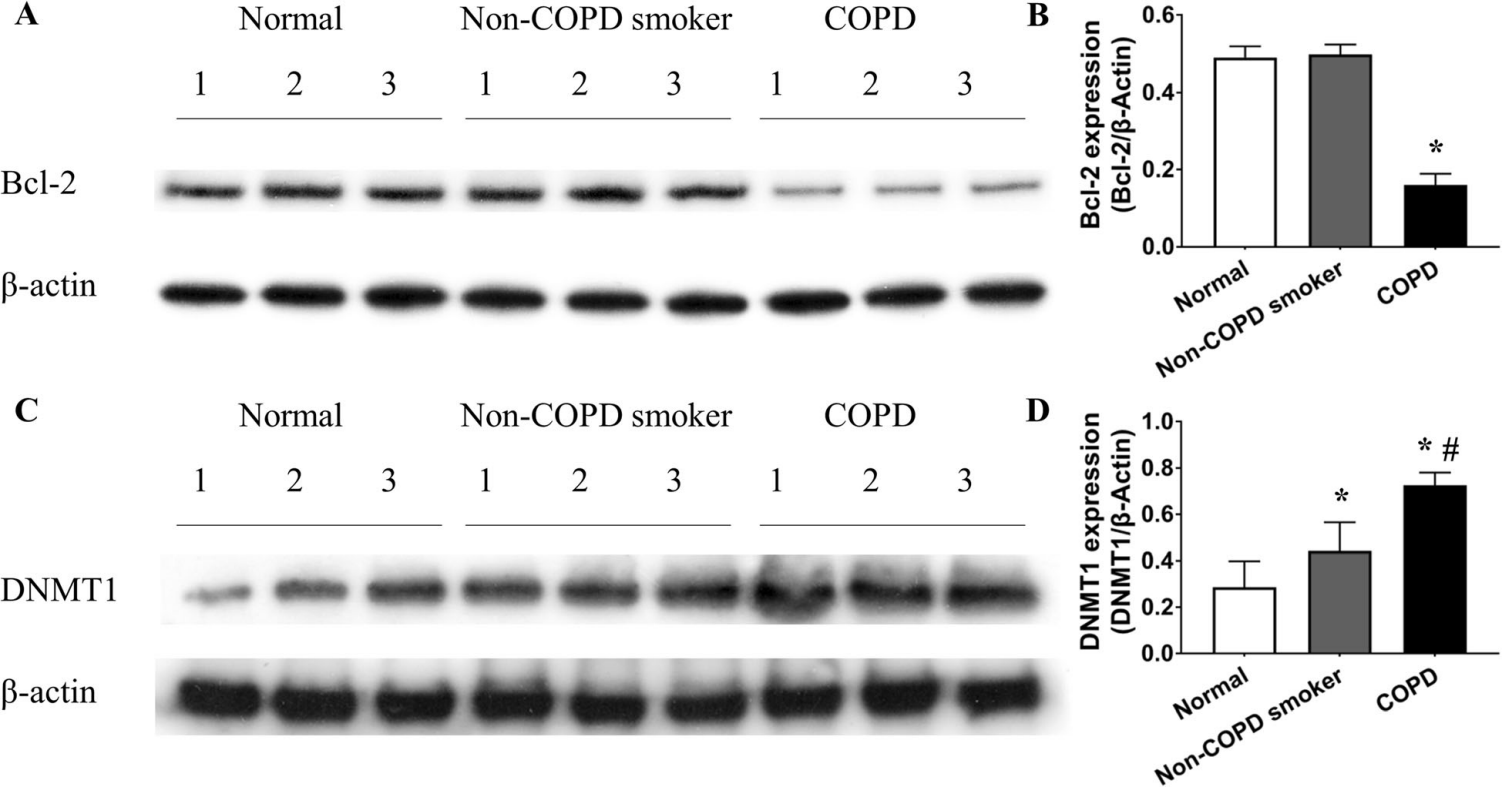
Bcl-2 and DNMT1 protein dysregulation in COPD patient lungs. **a** Lung tissues from normal (nonsmokers), non-COPD smokers and COPD patients were analyzed for Bcl-2 protein using immunoblotting. **b** The densitometric analysis showed that Bcl-2 expression was decreased in the lungs of COPD patients. Bcl-2 expression in the lung tissue of COPD patients was lower than that in the lung tissue of nonsmokers (0.16 ± 0.03 vs. 0.49 ± 0.03, *P <* 0.01 by one-way ANOVA and LSD test). **c** Lung tissues from nonsmokers, non-COPD smokers and COPD patients were analyzed for DNMT1 protein expression using immunoblotting. **d** The densitometric analysis showed that DNMT1 accumulated in the lungs of COPD patients. DNMT1 expression in the lung tissue from COPD patients was higher than that in the lung tissue from nonsmokers (0.73 ± 0.06 vs. 0.29 ± 0.11, *P <* 0.01 by one-way ANOVA and LSD test). Furthermore, the COPD group had higher DNMT1 expression than the non-COPD smoker group (0.73 ± 0.06 vs. 0.44 ± 0.12, *P <* 0.01 by one-way ANOVA and LSD test). *P < 0.05 vs. the nonsmoker group, ^#^*P* < 0.05 vs. the non-COPD smoker group. Data in (**b**) and (**d**) represent the mean ± SD

Lung tissues from nonsmokers, non-COPD smokers and COPD patients were analyzed for the expression of Bcl-2, a well-known apoptosis regulator, by western blotting. Bcl-2 protein in lung tissue from COPD patients was lower than that in lung tissue from nonsmokers (Fig. 3a, b). To discover whether DNA methylation is involved in COPD, DNMT1 protein, the DNA methyltransferase, was analyzed in lung tissues (Fig. 3c). Immunoblotting showed that DNMT1 expression in lung tissue from non-COPD smokers and COPD patients was higher than that in lung tissue from nonsmokers (0.44 ± 0.12 and 0.73 ± 0.06 vs. 0.29 ± 0.11, *P <* 0.01 by one-way ANOVA and LSD test, Fig. 3c, d). In addition, the COPD group presented higher DNMT1 expression than the non-COPD smoker group (*P <* 0.01 by one-way ANOVA and LSD test, Fig. 3c, d).

### Hypermethylation of the Bcl-2 promoter in COPD patients

**Fig. 4:**
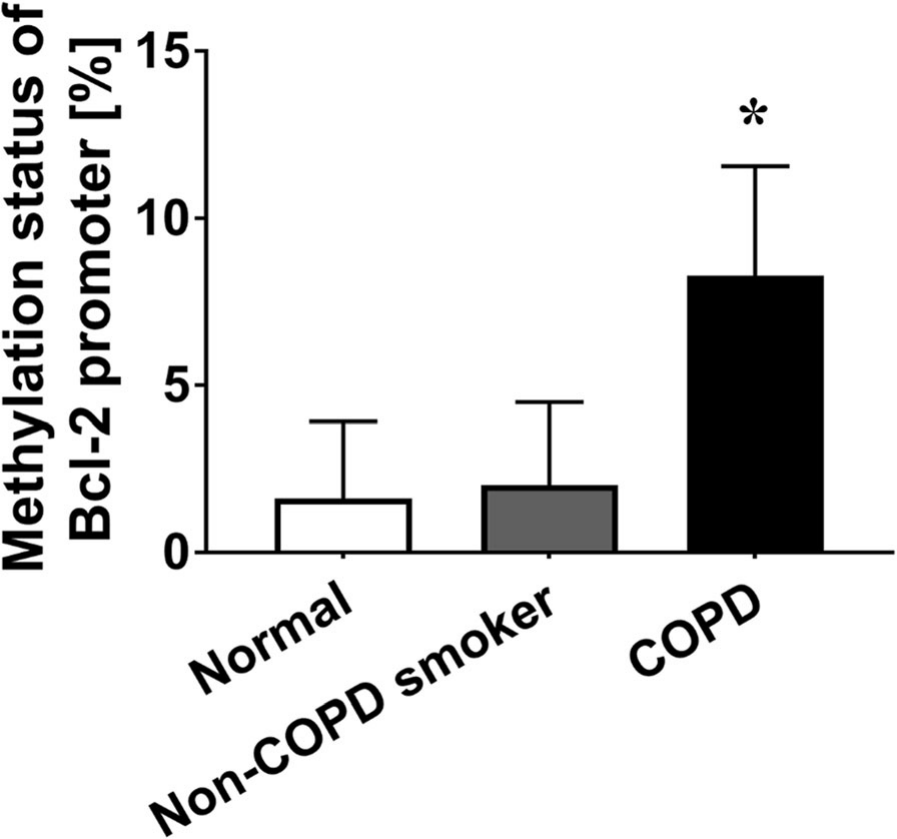
Hypermethylation of the Bcl-2 promoter in COPD patients. Compared to the normal (nonsmokers) group, the COPD group had an elevated Bcl-2 promoter methylation level (%) (1.62 ± 2.31 vs. 8.28 ± 3.29, *P <* 0.01 by one-way ANOVA and LSD test). There was no significant difference in the Bcl-2 promoter methylation level between the nonsmoker and non-COPD smoker groups (1.62 ± 2.31 vs. 2.02 ± 2.48, *P* = 0.756 by one-way ANOVA and LSD test). *P < 0.05 vs. Nonsmoker group. The data in the figure represent the mean ± SD

Given the higher expression of DNMT1 and lower expression of Bcl-2 in COPD patients than in healthy controls, we conducted bisulfite sequencing PCR (BSP) to detect the methylation status of the Bcl-2 promoter. The sequence results demonstrated that the COPD patients had an elevated level of Bcl-2 promoter methylation (Fig. 4). As the results of Bcl-2 protein detection, BSP showed that there was no significant difference in Bcl-2 methylation levels between the nonsmoker and non-COPD smoker groups (*P* = 0.756, Fig. 4). Considering the simultaneously increased DNMT1 expression and methylation level, it is possible to assume that the upregulated DNMT1 level leads to the hypermethylation of the Bcl-2 promoter in COPD patients.

### CS-induced oxidative stress participates in emphysema, pulmonary apoptosis and lung function damage

**Fig. 5:**
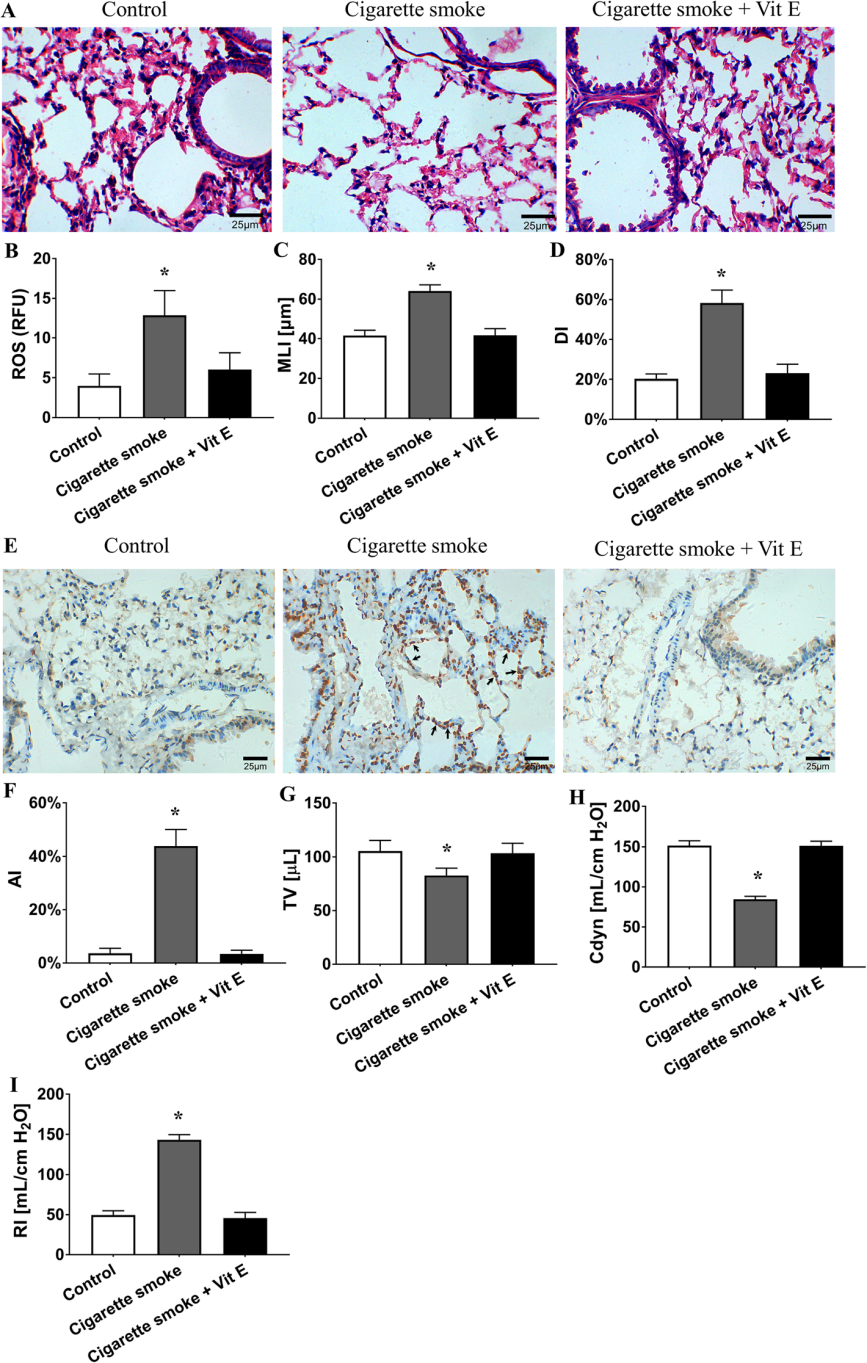
CS-induced oxidative stress is involved in emphysema, apoptosis, and lung function damage. **a** HE staining of lungs from the control, CS exposure and CS exposure with Vit E treatment groups (400×). **b** ROS levels in the lungs from the three groups. The CS exposure group had higher lung tissue ROS levels (RFU) than the control group (12.86 ± 3.12 vs. 4.00 ± 1.45, *P <* 0.01 by one-way ANOVA and LSD test). However, the CS exposure with Vit E feeding group showed a ROS level (RFU) similar to that of the control group (*P* = 0.07 by one-way ANOVA and LSD test). This indicates that antioxidant feeding could reverse the ROS level increase in CS exposed mice. **c**, **d** MLI and DI were calculated in the three groups. Compared to the control group, the CS exposure group had an increased MLI (μm) and DI (%) (41.58 ± 2.73 vs. 64.07 ± 3.12, *P <* 0.01 by one-way ANOVA and LSD test; 20.25 ± 2.39 vs. 58.21 ± 6.52, *P <* 0.01 by Kruskal-Wallis test). This demonstrates that the mice exhibited CS-induced emphysematous changes. In contrast, the CS exposure with Vit E treatment group showed MLI and DI values similar to those of the control group (*P* = 0.93 by one-way ANOVA and LSD test, *P* = 0.32 by Kruskal-Wallis test, respectively). **e** TUNEL staining in lung samples from the three groups (400×). → the TUNEL-positive nuclei. **f** AI was calculated in the three groups. Compared to the control group, the CS exposure group had an elevated AI (%) (3.65 ± 1.87 vs.43.90 ± 6.10, *P <* 0.01 by Kruskal-Wallis test). The CS plus Vit E group had an AI similar to that of the control group (*P* = 0.82 by Kruskal-Wallis test), (**g**), (**h**), (**i**) TV, Cdyn and RI in the three groups. The mice with CS exposure had a lower TV (μL) than the control mice (82.70 ± 6.93 vs. 105.50 ± 9.96, *P <* 0.01 by one-way ANOVA and LSD test). The CS exposure group also had worse Cdyn (mL/cm H_2_O) and RI (mL/cm H_2_O) values than the control mice (84.70 ± 3.65 vs. 151.6 ± 5.97 and 143.40 ± 6.24 vs. 49.6 ± 5.32, respectively, *P <* 0.01 by one-way ANOVA and LSD test). However, the CS plus Vit E treatment group exhibited similar TV, Cdyn and RI values as the control group (*P* = 0.63, *P* = 0.90 and *P* = 0.18 by one-way ANOVA and LSD test, respectively). These results reveal that antioxidant treatment alleviates the pulmonary morphological and functional damage caused by CS. *P < 0.05 vs. the control group. Data in the figure represent the mean ± SD

To confirm that oxidative stress contributes to COPD pathogenesis, we treated mice with CS and vitamin E (Vit E), one of the most common antioxidant reagents. Consistent with previous studies [13–15], we found that CS exposure caused increased ROS levels in lung tissue (Fig. 5a). Antioxidant feeding with Vit E prevented the increased ROS levels in CS exposed mice (Fig. 5a). Moreover, CS-treated mice exhibited emphysematous changes with aggravated MLI, DI and AI (Fig. 5b, c, d, and e). Coincidentally, the mice exposed to CS showed impaired pulmonary function, including deteriorated tidal volume (TV, mL), dynamic compliance (Cdyn, mL/cm H_2_O) and airway resistance (RI, cm H_2_O/mL/min; Fig. 5f, g). Interestingly, antioxidant treatment also alleviated the pulmonary morphological and functional damage caused by CS (Fig. 5b, c, d, e, f, and g). This finding suggests that CS-induced oxidative stress plays a role in emphysema, pulmonary apoptosis and lung function damage through oxidative stress.

### CS-induced oxidative stress is involved in the dysregulation of Bcl-2, cleaved caspase-3 and DNMT1 protein expression and DNA hypermethylation of the Bcl-2 promoter

**Fig. 6:**
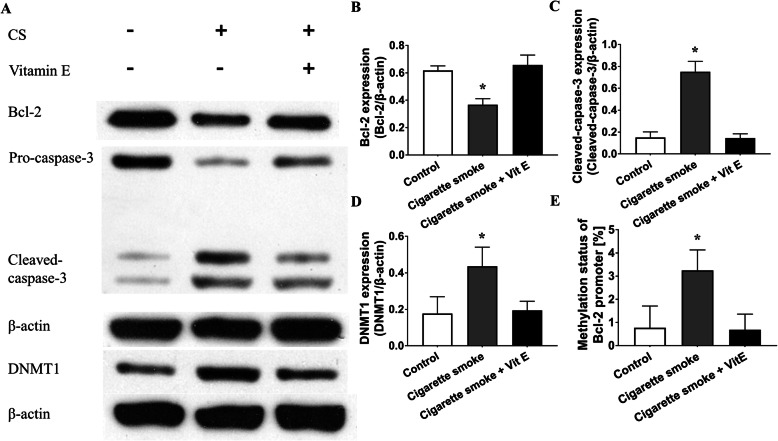
CS-induced oxidative stress is involved in the dysregulation of Bcl-2, cleaved caspase-3 and DNMT1 protein expression and the DNA hypermethylation of Bcl-2 promoter. **a** Immunoblotting was conducted in lungs from the control, CS exposure and CS exposure with Vit E treatment groups. **b** Densitometric analysis of Bcl-2 protein. Compared to the control group, the CS group exhibited decreased Bcl-2 protein level in the pulmonary tissue (0.62 ± 0.03 vs. 0.37 ± 0.05, *P <* 0.01 by one-way ANOVA and LSD test). However, the CS plus Vit E treatment group showed a Bcl-2 protein level similar to that of the control group (*P* = 0.10 by one-way ANOVA and LSD test). **c** The densitometric analysis of cleaved caspase-3. The expression of cleaved caspase-3 in the CS group was higher than that in the control group (0.75 ± 0.10 vs. 0.15 ± 0.05, *P <* 0.01 by Kruskal-Wallis test). In addition, the CS plus Vit E treatment group showed cleaved caspase-3 protein expression similar to that of the control group (*P* = 0.86 by Kruskal-Wallis test). **d** The densitometric analysis of DNMT1. Compared to the control group, the CS group had higher DNMT1 protein expression in the pulmonary tissue (0.18 ± 0.09 vs. 0.44 ± 0.10, *P <* 0.01 by Kruskal-Wallis test). In contrast, the CS plus Vit E treatment group showed DNMT1 protein expression similar to that of the control group (*P* = 0.96 by Kruskal-Wallis test). **e** DNA methylation level of the Bcl-2 promoter in the three groups. Compared to the control group, the CS group had increased Bcl-2 promoter methylation levels (%) in the lung (0.77 ± 0.94 vs. 3.25 ± 0.88, *P <* 0.01 by one-way ANOVA and LSD test). However, the CS plus Vit E treatment group showed a Bcl-2 promoter methylation level (%) similar to that of the control group (0.68 ± 0.67 vs. 0.77 ± 0.94, *P* = 0.79 by one-way ANOVA and LSD test). *P < 0.05 vs. the control group. The data in the figure represent the mean ± SD

Because of the increased apoptosis in COPD patients’ lungs, we detected the apoptosis regulator Bcl-2 and the apoptosis inducer caspase-3 in mouse models. Compared to the control mice, the CS exposed mice had lower Bcl-2 protein expression in the pulmonary tissue (0.62 ± 0.03 vs. 0.37 ± 0.05, *P <* 0.01 by one-way ANOVA and LSD test, Fig. 6a, b). Correspondingly, we also found that the level of cleaved caspase-3 protein in the CS group was higher than that in control group (0.75 ± 0.09 vs. 0.15 ± 0.05, *P <* 0.01 by the Kruskal-Wallis test, Fig. 6a, c). Given the hypermethylation of the Bcl-2 promoter in the lungs from COPD patients, we detected DNMT1 protein expression and the DNA methylation level of Bcl-2 in pulmonary tissue from mouse models. Immunoblotting showed that DNMT1 protein level was upregulated in the lungs from the CS group (Fig. 6a, d). BSP was subsequently performed, and showed that CS led to the hypermethylation of the Bcl-2 promoter (Fig. 6e). In contrast, the antioxidant reagent ameliorated the CS-induced dysregulation of the apoptosis-associated proteins Bcl-2 and cleaved caspase-3. Moreover, it prevented the alternation in DNMT1 protein level and the CS-induced epigenetic modification of Bcl-2 (Fig. 6a, b, c, d, and e). This finding indicates that CS-induced oxidative stress causes the dysregulation of apoptosis-associated proteins and gene methylation status, and ultimately initiates apoptosis.

### DNMT1 gene silencing or pharmacologic antagonism ameliorates CS-induced emphysema, pulmonary apoptosis, and hypermethylation of the Bcl-2 promoter without altering ROS levels

**Fig. 7:**
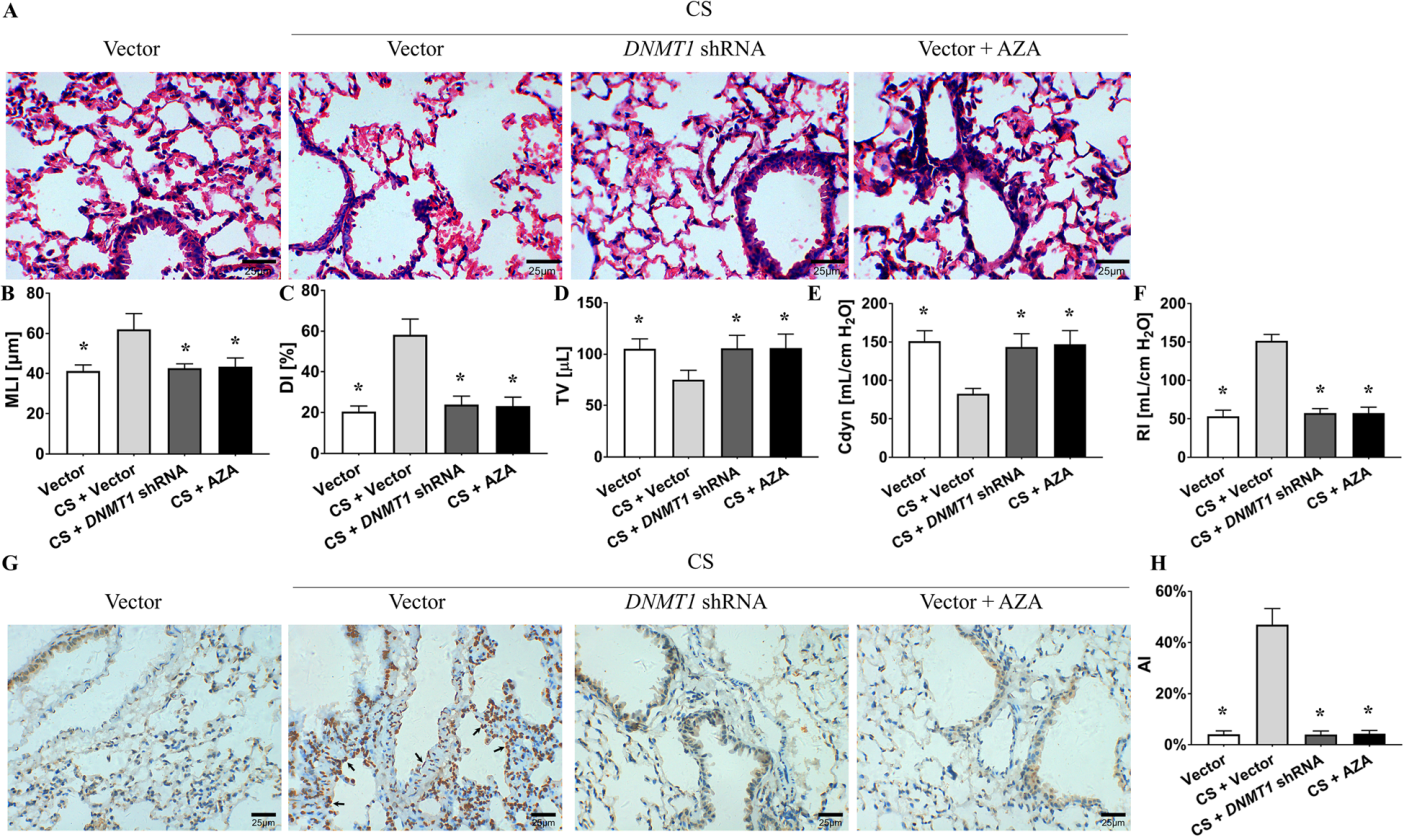
DNMT1 gene silencing or pharmacologic inhibition ameliorates CS-induced emphysema and pulmonary apoptosis without affecting ROS. **a** HE staining in the lungs of the control (vector), CS exposure (CS + vector), CS exposure plus DNMT1-silencing plasmid (CS + *DNMT1* shRNA) and CS exposure with AZA treatment (CS + AZA) groups. **b** ROS levels in lungs from the four groups. The CS exposure group had higher lung tissue ROS levels (RFU) than the control group (13.66 ± 3.04 vs. 3.80 ± 1.62, *P* < 0.01 by one-way ANOVA and LSD test). The CS plus *DNMT1* shRNA and CS plus AZA groups also had higher ROS levels than the control group (12.79 ± 2.54 and 11.76 ± 2.74 vs. 3.80 ± 1.62, *P <* 0.01 by one-way ANOVA and LSD test). This indicates that inhibiting DNMT1 does not reverse CS-induced ROS production and supports the hypothesis that DNMT1 could be a downstream factor in oxidative stress. **c**, **d** MLI and DI were calculated in the four groups. Compared to the CS group mice, the CS + *DNMT1* shRNA and CS + AZA mice had lower MLI and DI values (62.01 ± 7.87 vs. 42.60 ± 2.24 and 43.39 ± 4.37, 58.26 ± 7.69 vs. 23.89 ± 4.18 and 23.12 ± 4.36, respectively, *P <* 0.01 by Kruskal-Wallis test). **e**, **f** and **g** Lung function in the four groups. The results showed better TV, Cdyn and RI in the CS + *DNMT1* shRNA and CS + AZA mice than in the CS-exposed alone mice (105.56 ± 12.67 and 105.88 ± 13.68 vs. 75.11 ± 9.08, 143.44 ± 17.36 and 147.00 ± 17.85 vs. 82.33 ± 7.09, 57.33 ± 5.77 and 57.25 ± 7.76 vs. 151.44 ± 8.35, respectively, *P <* 0.01 by one-way ANOVA and LSD test). (H)TUNEL staining was conducted in lung tissues from the four groups. → the TUNEL-positive nuclei. **i** The AI was calculated in the four groups. TUNEL revealed less AI (%) in the CS + *DNMT1* shRNA and CS + AZA groups than in the CS group (4.01 ± 1.40 and 4.35 ± 1.24 vs. 47.02 ± 6.27, *P <* 0.01 by one-way ANOVA and LSD test). *P < 0.05 vs. the CS group, #*P <* 0.05 vs. the vector group. The data in the figure represent the mean ± SD

**Fig. 8:**
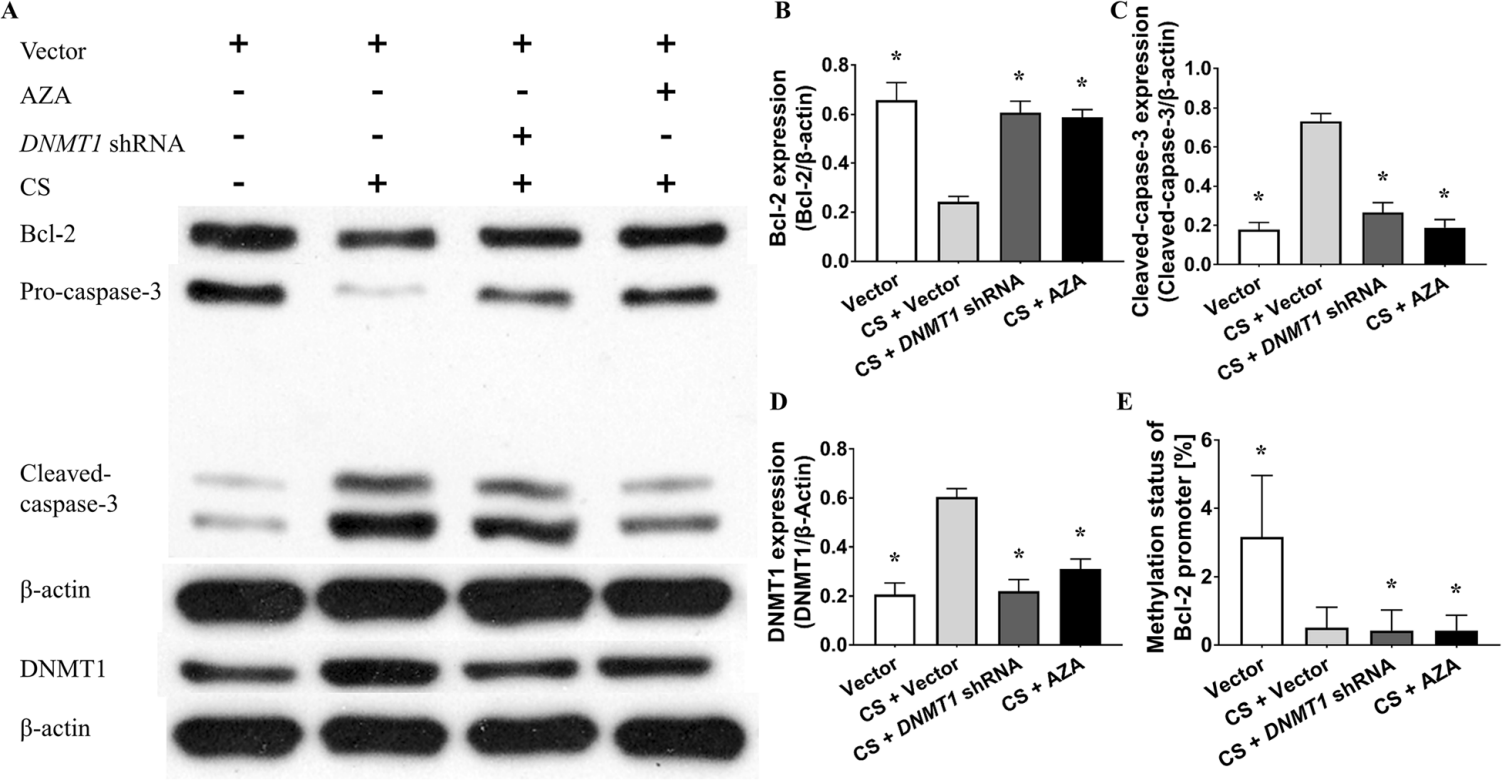
DNMT1 gene silencing or pharmacologic inhibition ameliorates the CS-induced dysregulation of Bcl-2 and cleaved caspase-3 protein expression and hypermethylation of the Bcl-2 promoter. **a** Immunoblotting was conducted using lungs from the vector, CS + vector, CS + *DNMT1* shRNA and CS + AZA groups. **b** Densitometric analysis of Bcl-2 protein. Compared to the CS group, the CS + *DNMT1* shRNA and CS + AZA groups had higher Bcl-2 expression (0.24 ± 0.03 vs. 0.61 ± 0.05 and 0.59 ± 0.03, *P <* 0.01 by one-way ANOVA). **c** The densitometric analysis of cleaved caspase-3. There was less cleaved caspase-3 in the CS + *DNMT1* shRNA and CS + AZA mice than in the CS mice (0.27 ± 0.05 and 0.19 ± 0.05 vs. 0.73 ± 0.04, *P <* 0.01 by one-way ANOVA). **d** The densitometric analysis of DNMT1. Compared to the CS group, the CS + *DNMT1* shRNA and CS + AZA groups expressed less DNMT1 (0.60 ± 0.03 vs. 0.22 ± 0.05 and 0.31 ± 0.04, *P <* 0.01 by one-way ANOVA). **e** Methylation status of Bcl-2 promoter in the lungs from the four groups. Compared to the CS group, the CS + *DNMT1* shRNA and CS + AZA groups had less Bcl-2 promoter methylation (%) in the lungs (3.95 ± 0.78 vs. 0.57 ± 0.60 and 0.47 ± 0.62, *P <* 0.01 by one-way ANOVA). These results indicate that DNMT1 gene silencing or inhibition prevented the downregulated Bcl-2 level, increased cleaved caspase-3 and hypermethylation in CS- exposed mice. *P < 0.05 vs. the CS group. The data in the figure represent the mean ± SD

Because of the elevated DNMT1 protein level and hypermethylation of Bcl-2 in the lung tissue of emphysema subjects, we tested whether modulation of the DNMT1 protein level or activity ameliorates emphysema, pulmonary apoptosis and Bcl-2 promoter hypermethylation in mouse models. The mice were depleted of DNMT1 intratracheally using a lentiviral delivery system [10^8^ plaque-forming units (pfu) per mouse] and subsequently exposed to CS. Compared to the mice from the control group, the mice from the CS group had a lower MLI and DI (Fig. 7a, c, d). The lung function test showed better TV and Cdyn in the CS + *DNMT1* shRNA treated mice than in the single CS-exposed mice (Fig. 7e, f, g). TUNEL staining also showed less pulmonary apoptosis in the CS + *DNMT1* shRNA group than in the single CS group (Fig. 7h, i). Immunoblotting revealed that there was higher Bcl-2 and lower cleaved caspase-3 protein levels in the CS + *DNMT1* shRNA group than in the CS group subjects (Fig. 8a, b, c). Furthermore, DNMT1 knockdown mice presented decreased methylation levels of the Bcl-2 promoter in the lungs (Fig. 8e). This result indicates that DNMT1 gene silencing prevented emphysema, pulmonary apoptosis, downregulated Bcl-2 expression, increased cleaved caspase-3 levels and Bcl-2 promoter hypermethylation in CS exposed mice.

5-Aza-2-deoxycytidine (AZA) is a well-known DNMT1 antagonist that can inhibit the activity and expression of DNMT1 in vivo and in vitro. We observed whether this antagonism protected CS exposed mice from pathological processes. As in our previous study [24], we found fewer emphysematous changes and less pulmonary apoptosis, including AI and cleaved caspase-3 expression, in the AZA plus CS treated mice than in the single CS-exposed models (Fig. 7, Fig. 8). Furthermore, the AZA plus CS treated mice showed lower DNMT1 expression and Bcl-2 promoter methylation levels, but higher Bcl-2 mRNA and protein expression than CS only exposed mice (Fig. 8e, Supplemental Fig. 1). The depletion of DNMT1 by shRNA or inhibition of DNMT1 with AZA markedly reduced lung function damage, emphysema, pulmonary apoptosis, and Bcl-2 expression, suggesting that CS exposure causes emphysema, pulmonary apoptosis and hypermethylation through DNMT1.

To determine whether DNMT1 controls oxidative stress via feedback, we observed ROS levels after DNMT1 gene silencing or pharmacologic inhibition. Interestingly, mice with DNMT1 knockdown still had higher ROS levels than control mice (Fig. 7b). This finding implies that inhibiting DNMT1 does not reverse CS-induced oxidative stress and supports the hypothesis that DNMT1 could be a downstream factor in oxidative stress.

## Discussion

In both human and animal experiments, COPD subjects were found to have emphysematous alterations, confirming that emphysema is one of the pathological characteristics of COPD. In addition, the thickened alveolar septum in non-COPD smokers suggests that CS might lead to alveolar inflammation. However, not all smokers develop COPD, indicating that there are mechanisms of this disease other than direct CS-induced destruction or inflammation.

Oxidative stress is an imbalance between oxidants and antioxidants in favor of oxidants [26]. Molecular oxygen, known as ROS, such as superoxide and hydroxyl radicals, alpha leukotrienes, and interleukins are the most pro-inflammatory factors in organisms [27]. ROS are one of the most common causes of cell death and disease processes, including pulmonary apoptosis and COPD [27, 28]. Consistent with previous studies [27, 28], this study provides evidence that there is increased ROS production in both COPD patients and mouse models. Furthermore, antioxidant treatment improved the dysregulation of lung function, apoptosis-associated proteins and apoptosis. These results indicate that oxidative stress induces pulmonary apoptosis and contributes to COPD pathogenesis.

Bcl-2 is a widely accepted antiapoptotic regulator that maintains the mitochondrial membrane and controls the activation of the caspase family [29, 30]. Our results demonstrated that Bcl-2 expression was significantly lower in COPD patients than in non-COPD smokers. Interestingly, Bai et al. [31] found that Bcl-2 expression was similar between emphysematous smokers and emphysematous patients. Almost all non-COPD smokers in our study did not experience emphysema, suggesting that Bcl-2 might specifically be involved in emphysema rather than other pathogenic processes of COPD. Our results showed that CS exposure decreased Bcl-2 expression and increased cleaved caspase-3 levels. Antioxidative treatment improved pulmonary apoptosis and downregulated Bcl-2 expression in CS-exposed mice. Given the essential role Bcl-2 plays in apoptosis, our results suggest that oxidative stress induces apoptosis and even COPD process though Bcl-2.

Methylation of the promoter, which could attach methyl groups to cytosine bases adjacent to guanine (CpG sites), is an emerging and essential pretranscriptional regulation mechanism. There is a CpG island in the promoter of both human and mouse Bcl-2 [32], which is rich in CpG sites and has the potential to be methylated. In addition, previous studies found that the hypermethylation of Bcl-2 promoter inhibits protein expression [33, 34]. The attractive characteristics of DNA methylation are heterogeneity and propagation. The heterogeneity of methylation reveals the differences in methylation status in the same genetic background, even in identical monozygotic twins. The heterogeneity might explain the differences in the morbidity of some diseases in parallel populations (i.e. COPD) [35, 36]. On the other hand, once methylation is initiated, it stably propagates over the lifetime, leading to persistent repression of gene expression [37]. Due to the ongoing progression of COPD after cigarette smoking cessation, we assume that the persistence of methylation might be involved in COPD. The sequencing results demonstrated that CS exposure increased the methylation of the Bcl-2 promoter. Antioxidant treatment prevented the hypermethylation of the Bcl-2 promoter in CS-exposed mice, suggesting that CS-induced oxidative stress is involved in the hypermethylation of the Bcl-2 promoter.

This methylation process is mostly determined by a family of DNA methyltransferase enzymes (DNMTs). DNMT1 is one of these enzymes, and it de novo methylates DNA and maintains the methylation status during replication [25]. Because of the increased methylation status of Bcl-2 in COPD patients and mouse models, we detected DNMT1 protein expression. Immunoblotting was conducted in antioxidant fed mouse models and confirmed that CS-induced ROS increased DNMT1 level. Based on this result, we assumed that CS-induced ROS or elevated oxidative stress might lead to increased Bcl-2 methylation status and pulmonary apoptosis through DNMT1. To test this hypothesis, we pretreated mice with *DNMT1* shRNA or AZA, a DNMT1 antagonist, before exposing them to CS. Finally, the results showed that DNMT1 gene silencing or pharmacological inhibition partly improved the dysregulated expression of Bcl-2, hypermethylation of the Bcl-2 promoter and apoptosis induced by CS exposure. Although the result could not absolutely exclude a potential effect of the post-translational process on modification of Bcl-2, it could partly support that DNMT1 and the methylation process regulate Bcl-2 expression. Moreover, DNMT1 knockdown did not reverse CS-induced ROS production, implying that DNMT1 does not directly regulate or control ROS production and might be a downstream factor of ROS. These results support the hypothesis that CS-induced ROS or increased oxidative stress lead to increased Bcl-2 promoter methylation and pulmonary apoptosis through DNMT1.

Finally, we found that DNMT1 gene silencing or pharmacologic inhibition prevented the dysregulation of pulmonary apoptosis, the emphysematous manifestations and lung function damage. Currently, COPD treatments in the real world might relieve some symptoms and acute exacerbation. However, there is no evidence that these treatments were effective enough to improve lung function in COPD patients [38]. This work might reveal a potential strategy for COPD prevention or treatment, as DNMT1 might be a potential target for new medication to prevent lung function damage.

There are some limitations to our research. This work investigated only the oxidant burden mediating the dysregulation of DNMT1, methylation and apoptosis, but did not detect antioxidants. According to the wide range of DNMT target genes [39], it is possible that aberrant DNMT1 expression might cause the hypermethylation of antioxidant genes and downregulation of antioxidant production. Additionally, this work found that non-COPD smokers had lower DNMT1 expression than COPD smokers. We hypothesize that DNMT1-induced hypermethylation of the Bcl-2 promoter is dose-dependent, or that there might be another protective mechanism in non-COPD smokers. To determine whether DNMT1 is involved in cell death by directly impeding antioxidant production or other protective mechanisms, our group will conduct a genome-wide analysis of DNA methylation in CS- or oxidant-exposed subjects. Our previous studies [13, 40] found that not only epithelial cells, but also endothelial cells were involved in emphysema. How epithelial and endothelial cells participate in the pathogenesis of COPD and whether there is an interaction between epithelial and endothelial cells are interesting and complicated. Therefore, the cellular mechanism will be discussed in our further study.

## Conclusion

In conclusion, this study shows that CS-induced oxidative stress mediates pulmonary apoptosis and the epigenetic modification of Bcl-2 via DNMT1. Furthermore, the findings support that the oxidative stress mechanism participates in COPD pathogenesis and that DNMT1 is involved in the COPD process.

## Methods

### Human samples

The study was approved and supervised by the Medical Research Ethics Committee of the Second Xiangya Hospital, Central South University. All participants fully understood the information files. Informed consent was obtained from all participants. All experiments were performed in accordance with the relevant guidelines and regulations.

Human lung samples were obtained from subjects who underwent peripheral solitary pulmonary nodule or pulmonary lesion excision at the Second Xiangya Hospital of Central South University. All specimens were sampled from the margin of the excised lung tissue, at least 5 cm away from the lesion location [41]. The subjects were divided into 3 groups: one group is COPD patients, the second group included smokers without COPD, and the third group included nonsmokers without COPD. COPD patients were diagnosed by following the Global Initiative for Chronic Obstructive Lung Disease (GOLD) 2014 criteria. Smokers were defined as subjects who had a history of at least 20 pack-years of cigarette smoking [2].

Samples were immediately frozen in liquid nitrogen and stored at − 80 °C until use. For immunostaining and TUNEL staining, tissue blocks were fixed in 10% formalin for at least 24 h. After being fixed, each tissue block was embedded in paraffin, and 3.5-μm-thick sections were cut by following routine procedures. The other lung tissues were homogenized in a buffer containing 50 mmol/L N-2-hydroxyethylpiperazine-N′-ethane sulfonic acid, 1 mmol/L dithiothreitol, 0.1% Triton X-100, and 10% glycerol immediately after being harvested. The supernatant was separated by two cycles of centrifugation at 1000×g for 10 min. The protein concentration was determined via the bicinchoninic acid (BCA) protein assay (Pierce, USA).

### Animal experiments

This animal protocol was approved by the Ethics Committee of the Second Xiangya Hospital, Central South University. C57BL/6 J mice (6 weeks old, male) underwent two experiments. The first experiment divided the mice into 3 groups (*n* = 10 each group). The control group was exposed to normal air from 8 to 20 weeks old, and the CS group was exposed to CS twice daily during the same period [42]. The last group was the Vit E plus CS exposure group, which was administered Vit E (100 mg/kg) orally daily from 7 to 20 weeks old and exposed to CS from 8 to 20 weeks old. The second step was conducted to assess whether DNMT1 plays a role in the pathogenesis of COPD. There were 4 groups (*n* = 10 each group) of mice in this step. The control group was treated with lenti*-empty* (10^8^ pfu per mouse, once a week, intratracheally) at 6 and 7 weeks old. The CS group was also treated with lenti*-empty* (10^8^ pfu per mouse, once a week, intratracheally) at 6 and 7 weeks old, and then exposed to CS from 8 to 20 weeks old. One mouse in the CS group died during the experiment. The last two groups were administrated lenti*-DNMT1* shRNA (10^8^ pfu per mouse, once a week, intratracheally) at 6 and 7 weeks old, or lenti-empty (10^8^ pfu per mouse, once a week, intratracheally) plus AZA (5 mg per kg, once a week, intraperitoneally) at 6 weeks old [19]. Both of the above two groups were exposed to CS for 12 weeks from 8 weeks old, and labeled as the CS + *DNMT1* shRNA and CS + AZA groups. One CS + *DNMT1* shRNA mouse died during the experiment, and two CS + AZA mice died during the experiment.

Consistent with the human samples, the left lung tissues of mice were inflated with 10% formalin at a constant pressure of 25 cm H_2_O for 24 h and subsequently fixed and embedded. The protein was extracted by following the same protocol as that used for the human samples.

### Pulmonary function

Mice were anesthetized by intraperitoneal injection of chloral hydrate (3 ml/kg). As previous study [43], pulmonary function was measured in intubated mice using Plethysmograph (Buxco Research Systems, USA). Tidal volume (TV, mL), dynamic compliance (Cdyn, mL/cmH2O), and airway resistance (RI, cmH2O/mL/min) were measured in each sample.

### Morphology and apoptosis assessment

The sections of lungs were stained with hematoxylin and eosin (HE). Emphysema was quantified based on the degree of alveolar destruction, determined through measuring the MAST, MLI and DI. MAST was assessed by averaging 400 measurements per 10 non-overlapping fields in sections by microscopy at 400× magnification [44]. MLI was assessed by dividing the length of a line drawn across the section by the total number of intercepts encountered in 36 lines per sample, and 10 random fields per sample were observed by microscopy at a magnification of 100× [14, 45]. DI was assessed by dividing the number of destroyed alveoli by the total number of alveoli counted, and an average of 5 different sections was observed in each sample under microscopy at a magnification of 100×. Alveolar destruction alveoli was defined on the basis of the following criteria: at least 2 alveolar wall defects, at least 2 intraluminal parenchymal rags in alveolar ducts, clearly abnormal morphology, or classic emphysematous changes in the lung [46].

TUNEL staining was performed to estimate the apoptosis level in the lung tissue with an in-situ apoptosis detection kit (Nanjing Keygen Biotech, China). The AI was determined in lung tissue from each subject to detect the apoptosis status of the lung parenchyma, and was calculated as the percentage of TUNEL-positive nuclei out of a total of more than 3000 nuclei randomly at 400× magnification [40]. Both morphology and apoptosis assessments were observed repeatedly by 3 pathologists.

### Reactive oxygen species (ROS) detection

The total ROS in the lung was detected using a dichlorofluorescein diacetate (DCFH-DA) kit (Genmed Scientifics, USA) [47]. In according with the kit instructions, the tissue was homogenized. The DCFH-DA signal was measured with a Molecular Devices SPECTRAMAX M5 fluorimeter (490 nm excitation and 530 nm emission).

### Immunoblotting

The extracted protein was separated on an SDS-PAGE gel (Beyotime, China) and transferred to a nitrocellulose (NC) membrane (Millipore, USA). Following protein transfer, the membrane was blocked with 5% nonfat milk, and washed. Then, the membrane was incubated overnight with antibodies against DNMT1 (1/1000, Proteintech, USA for human tissue, 3 μg/mL, Abcam, UK for mouse tissue), Bcl-2 (1/1000, Cell Signaling Technology, USA), caspase-3 (1/1000, Cell Signaling Technology, USA) and β-actin (1/4000 for human tissue, 1/5000 for mouse tissue, Proteintech, USA) [33, 41]. After being washed four times with PBST, the membrane was incubated with HRP-conjugated IgG (Jackson Immuno Research Laboratories, USA) for 1 h at room temperature. Protein band detection was performed using an ECL kit (Thermo, USA), and films were developed and fixed by a developer and fixer kit (Beyotime, China). The blots were quantitated with Quality-one software (Bio-Rad Laboratories, CA).

### Real time reverse transcriptase-polymerase chain reaction

RNA was extracted as previously described [48]. RNA was reverse-transcribed using the PrimeScript RT reagent kit (Takara, China), and assayed using SYBR (Takara, China) following the manufacturer’s instructions. All of the primers were obtained from Sangon Shanghai, China (Supplemental Table 1). Real time polymerase chain reaction (PCR) was carried out on the Step-one ABI Real-time RT-PCR system. All mRNA expression values were presented relative to β-actin.

### Bisulfite sequencing PCR (BSP) assay

The Bcl-2 promoter in human was determined to range from − 3000 to − 70 bp by the Transcriptional Regulatory Element Database from Cold Spring Harbor (http://rulai.cshl.edu/cgi-bin/TRED/tred.cgi?process=promInfo&pid=19717). The Bcl-2 promoter in mouse was searched in the Transcriptional Regulator Element Database (accession number 46672, NM 009741). The CpG island in the promoter (− 1867 to − 1541 bp) was detected using the UCSC Genome Browser, and the methylation status was analyzed using BSP. Primers (Supplementary Table 2) for BSP were designed through MethPrimer (http://www.urogene.org/methprimer/), and then were blasted and confirmed using methBLAST.

A Genomic DNA Extraction kit (Takara, Japan) was used to extract DNA from the lungs. The bisulfite conversion of DNA was performed with an EpiTect Bisulfite Kit (QIAGEN, Netherlands) by following the manufacturer’s instructions. Subsequently, nested PCR was performed on the bisulfate-modification samples.

### Plasmid construction

The mouse DNMT1 sequence was obtained from GenBank (https://www.ncbi.nlm.nih.gov/genbank/). The DNMT1 sequence was cloned with a BD In-Fusion PCR Cloning Kit (BD Biosciences, US), and the construction of the lentiviral vector was outsourced to VectorBuilder (Cyagen Biosciences Inc., CA). Through screening, our group chose the *DNMT1* shRNA*,* targeting the sequence TCGACCTGGTTTGATACTTAT.

### Statistical analysis

A software package (SPSS 16.0; Statistical Product and Service Solutions, USA) was used to perform all statistical analyses. The values are described as the means ± SD. One-way ANOVA and Kruskal-Wallis tests were performed to evaluate each group of data. *P* values less than 0.05 were considered statistically significant.

## Supplementary information

**Additional file 1: Table S1.** Primers for real time-PCR. **Table S2.** Primers for BSP. **Table S3.** Primary data of patients. **Figure S1.** DNMT1 gene silencing or pharmacologic inhibition prevents the CS-induced dysregulation of Bcl-2 mRNA expression. Real-time PCR was conducted using lungs from the vector, CS + vector, CS + *DNMT1* shRNA and CS + AZA groups. *P < 0.01 vs. the CS group by one-way ANOVA. The data in figure represent the mean ± SD.

## Data Availability

We would like to share part of our data, because some of our date will be used in future research.

## Abbreviations

AI: apoptosis index
ANOVA: analysis of variance
AZA: 5-Aza-2-deoxycytidine
BCA: bicinchoninic acid
Bcl-2: B-cell lymphoma-2
BSP: bisulfite sequencing PCR
Cdyn: dynamic compliance
COPD: chronic obstructive pulmonary disease
CS: cigarette smoke
DCFH-DA: dichlorofluorescein diacetate
DI: destructive index
DNA: deoxyribose nucleic acid
DNMT1: DNA methyltransferase 1
ECL: efficient chemiluminescence
GOLD: the Global Initiative for Chronic Obstructive Lung Disease
HRP: horseradish peroxidase
LSD: least significant difference
MAST: mean alveolar septal thickness
MLI: mean linear intercept
NC: nitrocellulose
PBST: phosphate buffered saline and Tween-20
PCR: polymerase chain reaction
RI: inspiratory resistance
ROS: reactive oxygen species
SDS-PAGE: SDS-polyacrylamide gel electrophoresis
TUNEL: TdT-mediated dUTP nick-end labeling
TV: tidal volume
UCSC: University of California, Santa Cruz
