# Supplementary material for: Oxidative stress mediates the apoptosis and epigenetic modification of the Bcl-2 promoter via DNMT1 in a cigarette smoke-induced emphysema model
Source: Respir Res. 2020 Sep 3;21:229. doi: 10.1186/s12931-020-01495-w (PMC7469342; doi:10.1186/s12931-020-01495-w)
Supplement: Supplementary file 1 — Additional file 1: Table S1. Primers for real time-PCR. Table S2. Primers for BSP. Table S3. Primary data of patients. Figure S1. DNMT1 gene silencing or pharmacologic inhibition prevents the CS-induced dysregulation of Bcl-2 mRNA expression. Real-time PCR was conducted using lungs from the vector, CS + vector, CS + DNMT1 shRNA and CS + AZA groups. *P < 0.01 vs. the CS group by one-way ANOVA. The data in figure represent the mean ± SD. [file 12931_2020_1495_MOESM1_ESM.docx]

Supplemental Table 1. Primers for real time-PCR

| Name | Primer sequence | |
| --- | --- | --- |
| β-actin | Forward | 5’- CATCCTGCGTCTGGACCTGG -3’ |
|  | Reverse | 5’- TAATGTCACGCACGATTTCC -3’ |
| Bcl-2 | Forward | 5’- TGACTTCTCTCGTCGCTACCGT-3’ |
|  | Reverse | 5’- CCTGAAGAGTTCCTCCACCACC -3’ |

Supplemental Table 2. Primers for BSP

| Name | Primer sequence | |
| --- | --- | --- |
| (Human) Bcl-2 primer | Forward | 5’- AGGAATTGGAATAAAAATTTTTTGTATT -3’ |
|  | Reverse | 5’- ACAACTTATAATAAATATACTTCATCACTA -3’ |
| (Mice) Bcl-2 outer primer | Forward | 5’- GAAAGGGTTTATTGGATTGTGT -3’ |
|  | Reverse | 5’- AAAAAAAAAAAAAAAACCCTCCT -3’ |
| (Mice) Bcl-2inner primer | Forward | 5’- GGTTTATGTGATTTGTATATGTTATATAGA -3’ |
|  | Reverse | 5’- AAAAAAAACCCTCCTCTAAAC -3’ |

Supplemental Table 3. Primary data of patients

| Item | Patients number | Nonsmoker | Smoker without COPD | COPD |
| --- | --- | --- | --- | --- |
| Age (year) | 1 | 45 | 56 | 55 |
|  | 2 | 59 | 52 | 57 |
|  | 3 | 65 | 66 | 59 |
|  | 4 | 72 | 67 | 65 |
|  | 5 | 55 | 53 | 55 |
|  | 6 | 53 | 59 | 60 |
|  | 7 | 48 | 57 | 69 |
|  | 8 | 69 | 51 | 50 |
|  | 9 | 40 | 56 | 49 |
| Smoking history (pack-year) | 1 | 0 | 32 | 30 |
|  | 2 | 0 | 25 | 32 |
|  | 3 | 0 | 40 | 45 |
|  | 4 | 0 | 36 | 60 |
|  | 5 | 0 | 45 | 50 |
|  | 6 | 0 | 60 | 60 |
|  | 7 | 0 | 48 | 35 |
|  | 8 | 0 | 57 | 20 |
|  | 9 | 0 | 20 | 24 |
| FEV_1_/Pre (%) | 1 | 86 | 88 | 70 |
|  | 2 | 79 | 89 | 75 |
|  | 3 | 89 | 77 | 69 |
|  | 4 | 85 | 90 | 68 |
|  | 5 | 83 | 89 | 77 |
|  | 6 | 84 | 86 | 75 |
|  | 7 | 82 | 86 | 65 |
|  | 8 | 86 | 83 | 73 |
|  | 9 | 92 | 82 | 74 |
| FEV_1_/FVC (%) | 1 | 85 | 89 | 63 |
|  | 2 | 79 | 88 | 66 |
|  | 3 | 75 | 78 | 60 |
|  | 4 | 74 | 73 | 69 |
|  | 5 | 80 | 77 | 59 |
|  | 6 | 78 | 79 | 61 |
|  | 7 | 90 | 76 | 68 |
|  | 8 | 88 | 87 | 62 |
|  | 9 | 83 | 83 | 63 |


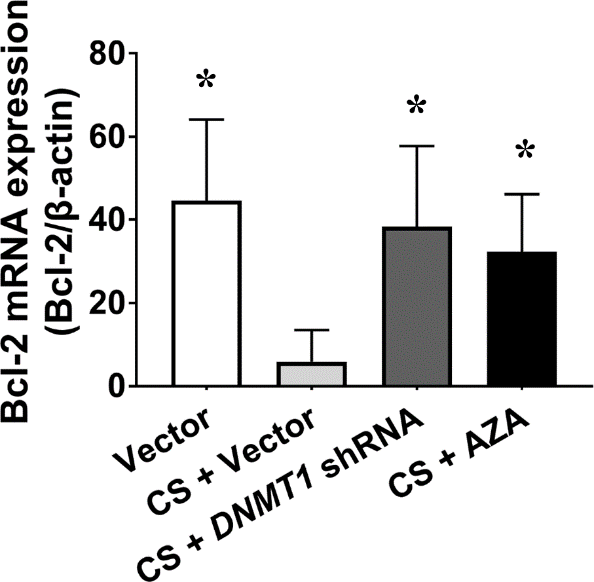


Supplemental Figure 1. DNMT1 gene silencing or pharmacologic inhibition prevents the CS-induced dysregulation of Bcl-2 mRNA expression.

Real-time PCR was conducted using lungs from the vector, CS + vector, CS + *DNMT1* shRNA and CS+AZA groups. *P<0.01 vs. the CS group by one-way ANOVA. The data in figure represent the mean ± SD.
